# Investigating the infant gut microbiota in developing countries: worldwide metagenomic meta‐analysis involving infants living in sub‐urban areas of Côte d'Ivoire

**DOI:** 10.1111/1758-2229.12960

**Published:** 2021-06-21

**Authors:** Federico Fontana, Leonardo Mancabelli, Gabriele Andrea Lugli, Chiara Taracchini, Giulia Alessandri, Giulia Longhi, Rosaria Anzalone, Alice Viappiani, Roch Famo, Marc Brognan, Kouamé Hervé Micondo, Francesca Turroni, Marco Ventura, Rossella D'Alfonso, Christian Milani

**Affiliations:** ^1^ Laboratory of Probiogenomics, Department of Chemistry, Life Sciences, and Environmental Sustainability University of Parma Parma Italy; ^2^ GenProbio srl Parma Italy; ^3^ Centre Médical Don Orione Anyama Anyama Côte d'Ivoire; ^4^ Microbiome Research Hub University of Parma Parma Italy; ^5^ Pediatric Service of Hospital Military D'Abidjan Abidjan Côte d'Ivoire; ^6^ Department of Systems Medicine University of Rome Tor Vergata Rome Italy

## Abstract

In recent decades, infants' gut microbiota has aroused constant scientific interest, primarily due to early‐ and long‐term repercussions on the host health. In this context, nutritional challenges such as those found in less developed countries can influence infants' gut microbiota development, thus generating potentially critical health outcomes. However, comprehensive investigations regarding species‐level differences in the infant gut microbiota's composition between urbanized and rural countries are still missing. In this study, 16S rRNA and Shallow Shotgun metagenomics sequencing were exploited to dissect the microbial community's species‐level composition of 11 faecal samples collected from infants living in a semi‐urban area of Sub‐Saharan Africa, i.e. Côte d'Ivoire. Moreover, the generated data were coupled with those retrieved from public available metagenomic repositories, including two rural communities and 13 urban communities of industrialized countries. The meta‐analysis led to the identification of Infant Species Community States Type (ISCSTs) and microbial species covariances, which were exploited to reveal key signatures of infants living in rural and semi‐urban societies. Remarkably, analysis of rural and semi‐urban datasets revealed shifts from ISCSTs prevalent in urbanized populations with putative health implications. Thus, indicating the need for population‐wide investigations aimed to define the factors determining such potentially harmful gut microbial communities' signatures.

## Introduction

The gastrointestinal tract is commonly colonized by a large variety of microorganisms, which coexist with the host and constitute a complex ecosystem shaped by external stimuli, such as nutrition and drug intake. Human gut microbiota is known to influence the host's health status in different ways (Adak and Khan, 2019). In such a context, it can exert positive influences, but this requires that the microbiota is under a homeostatic equilibrium, or it can negatively affect human health in perturbation of balance between bacterial species, known as dysbiosis that potentially favours pathological conditions (Nagpal and Yamashiro, 2018; Turroni *et al*., 2020). Furthermore, early dysbiosis in infants can alter the development of the adult gut microbiota, leading to unpleasant mid and long‐term effects (Savino *et al*., 2004; Schirbel and Fiocchi, 2011; Marchesi *et al*., 2016).

Therefore, it is essential to profile and monitor the bacterial communities to identify significant shifts predisposing to dysbiotic conditions. It is crucial to extend our investigations regarding the infant gut microbiota composition within the first year of life, which is considered a critical window of time to understand the factors protecting later health (Milani *et al*. 2017a; Cukrowska *et al*. 2020a). The first major factor impacting the early stages of infant gut microbiota development is the delivery method, i.e. Natural and C‐Section (Rutayisire *et al*., 2016). Feeding with breast or formula milk modulates the intestinal microbial composition of the newborn (De Leoz *et al*., 2015; Le Doare *et al*., 2018; Lugli *et al*., 2020). During weaning, which generally occurs around 6 months after birth, the intestinal microbiota's development is driven by the bacterial colonization from the mother, the environment and the diet (Matamoros *et al*., 2013; Gentile and Weir, 2018). The crucial step for intestinal bacterial development is determined by changes in diet. Indeed, novel micro‐ and macronutrients are available and new substrates start the selection of new bacterial strains based on their metabolic ability (Yadav *et al*., 2018).

Recently, 12 highly recurrent gut microbial profile structures, known as Infants Community State types (ICSTs), have been identified with genus‐level accuracy within the first year of life, of which five associated with lactation and seven with post‐weaning gut microbiota development (Mancabelli *et al*., 2020). However, the ICSTs identified in the available scientific literature are based on samples collected from infants living in urbanized and sub‐urbanized countries. Therefore rural or semi‐urban countries have not been assayed in this analysis. Moreover, the latter studies relied on 16S rRNA gene microbial profiling data, resulting in genus‐level accuracy.

In this study, a comprehensive meta‐analysis of 1109 shotgun metagenomics datasets of infant's faecal samples collected and sequenced in this study or previously released in the framework of published scientific literature was selected, representing a range of geographical regions including 13 urbanized area, two rural areas from Malawi and sub‐Saharan Africa as well as one sub‐urban area from Côte d'Ivoire collected and submitted to sequencing in this study. The use of shotgun metagenomics data resulted in the identification of novel Community State Types at the species level resolution (Laudadio *et al*., 2018). These new CSTs, defined in this study as Infants Species Community State Types (ISCSTs), along with species‐level profiling covariance analyses, were exploited to explain how the faecal microbial profiles of the rural and semi‐urban communities can be related to the most common ISCSTs observed in urbanized communities.

## Results and discussion

### 16S rRNA genera profiling analysis of 11 infants from Côte d'Ivoire

In the framework of this study, 11 faecal samples of infants from Côte d'Ivoire with no reported health problems were collected between 10 and 75 days of life. Notably, these represent the first investigation of the gut microbiota community harboured by infants living in this developing country. Nine out of 11 of these infants were fed with a diet based on mother's milk, while only two were fed with a mixed formula (Table S1). In addition, 1098 faecal samples, corresponding to urbanized and rural infants aged between 1 day and 1 year, were selected from 16 different publicly available shotgun metagenomics datasets obtained by Illumina sequencing to cover both pre‐ and post‐weaning gut microbiota development (Table S1; Fig. S1).

A preliminary 16S rRNA profiling analysis, encompassing the 11 Côte d'Ivoire samples, was performed to obtain an overview of the gut microbiota composition at the genus level. The 16S rRNA profiling is a technique based on sequencing of a specific region of the bacterial ribosomal locus, i.e. 16S region, which allows obtaining an accurate bacterial taxonomic classification down to genus level (Fig. S2; Table S2) (Costea *et al*., 2017; Mancabelli *et al*., 2020). The results, consistently with those described in the so‐far published scientific literature, showed that the genera *Bacteroides*, *Bifidobacterium*, *Escherichia*, *Veillonella* and *Clostridium* were the dominant taxa in the 11 Côte d'Ivoire subjects, with an average relative abundance ranging from 7.42% to 18.63% (Table S2) (Decuypere *et al*., 2016; Laudadio *et al*., 2018).

Furthermore, prevalence analysis performed by considering microbial taxa with relative abundance >0.01% revealed that *Escherichia‐Shigella* and *Streptococcus* were present with a prevalence of 100%. At the same time, members of the *Bifidobacterium* genus, with documented host' health benefits (Milani *et al*., 2017), were present in 10 samples from Côte d'Ivoire while absent only in one sample, named 15‐BE (Table S2). This latter sample showed a high abundance of *Clostridium* and *Klebsiella* species, which are both correlated with an unhealthy status of the gut microbiota and remarkably this sample revealed the absence of *Bifidobacterium* (Table S2).

## Species‐level profiling through shallow shotgun metagenomics of Côte d'Ivoire infant gut microbiota

**Fig. 1:**
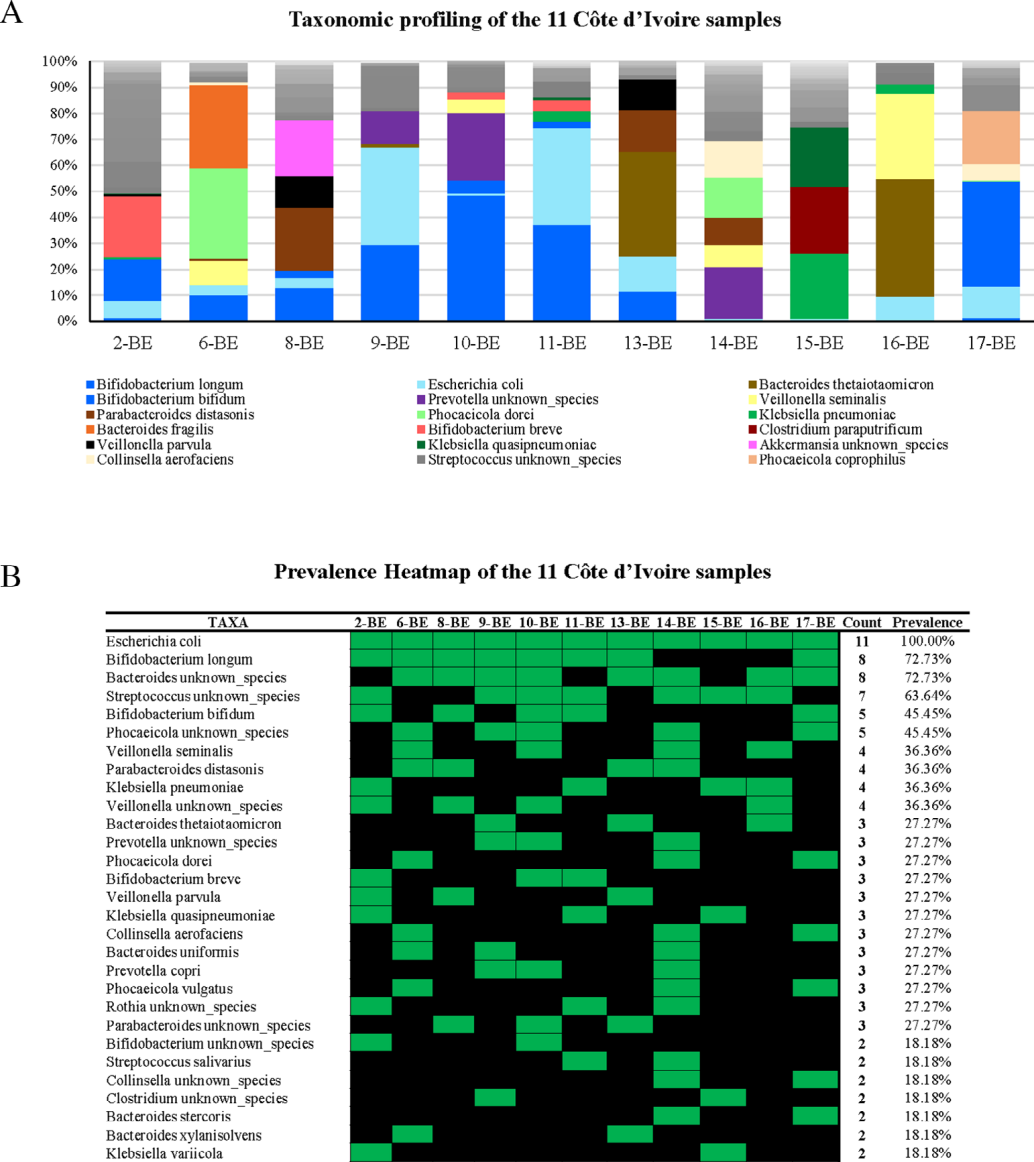
Taxonomic profiling of the 11 Côte d'Ivoire samples collected in this study. Panel A shows a bar plot representation of the taxonomic composition of the 11 Côte d'Ivoire infants' samples. Panel B reports a prevalence heatmap, showing taxa identified in at least two samples among the pool of 11 infants' samples.

Following the assessment of the genera‐level gut microbiota composition of the 11 Côte d'Ivoire samples, we re‐analysed all samples through the shallow shotgun metagenomics method to achieve bacterial taxonomic classification at species‐level (Hillmann *et al*., 2018). The above described 16S rRNA‐based profiles at the genus level were compared with those obtained through shallow‐shotgun metagenomics, showing a perfect match between them with high reliability (Fig. S2). Thus, shallow shotgun metagenomics analysis allowed us to properly decipher the intra‐genera complexity of the faecal microbial communities (Table S3; Fig. 1) (Hillmann *et al*., 2018).

Notably, the species‐level prevalence matrix showed that 29 bacterial species are shared by two or more samples, covering more than 80% of the total average compositions of each sample (Table S3; Fig. 1). Among these 29 species, three were shared by more than 40% of the samples (5 out of 11 samples at least), i.e. *Escherichia coli*, *Bifidobacterium longum* and *Bifidobacterium bifidum*, which also showed with high average abundance (between 6.05% and 13.88%) (Table S3; Fig. 1). Remarkably, these results confirm that members of the *Bifidobacterium* genus represent key early gut colonizers, along with *E*. *coli*, as previously observed for infants living in urbanized countries (Milani *et al*. 2017b).

## Meta‐analysis of species‐level gut microbiota profiles of 1098 infants from publicly available datasets

A collection of 1098 publicly available shotgun samples, corresponding to faecal samples of urbanized and rural infants aged between few days of life to 12 months, were retrieved (Table S1; Fig. S1). Upon the inclusion of the 11 Côte d'Ivoire shallow shotgun datasets, a total of 1109 samples were grouped by infants' age in 1–3 M category (*n* = 309, 0–3 months of life), 3–6 M category (*n* = 588, 3–6 months of life), 6–9 M category (*n* = 114, 6–9 months of life) and 9–12 M category (*n* = 133, 9–12 months of life) (Table S1). To prevent biases due to data analysis, all the shotgun metagenomics datasets were re‐analysed using METAnnotatorX (Milani *et al*., 2018). In detail, all the datasets were quality‐filtered and 100 000 quality‐filtered reads were exploited for the species‐level taxonomic reconstruction of the faecal microbiota composition through the shallow metagenomics approach (Hillmann *et al*., 2018).

The gut microbiota composition of the 1109 abovementioned metagenomics datasets was assessed (Table S4). Data retrieved showed that the most abundant species was *B*. *longum*, with an average abundance of 16.07%, followed by *Escherichia coli*, *Bifidobacterium breve* and *B*. *bifidum* with an average abundance of 7.60%, 6.18% and 5.96% respectively (Table S4). Moreover, *B*. *longum*, *B*. *breve* and *E*. *coli* were present in more than 50% of the infants, showing a prevalence of 73.67%, 64.20% and 50.86% respectively (Table S5). Remarkably, the latter taxa were also the most prevalent and abundant in infants from Côte d'Ivoire sequenced in this study (Table S3; Fig. 1). Thus, suggesting that host‐associated factors may be the main responsible in gut microbiota development, while environmental factors may participate in defining the wide range of interindividual variations.

**Fig. 2:**
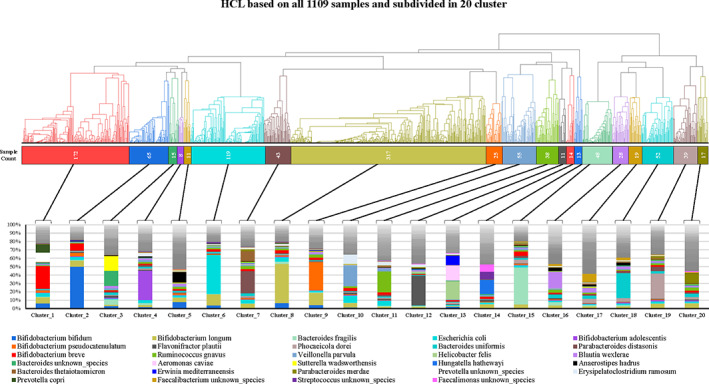
Average taxonomic composition of the predicted ISCST. The representation reports the average taxonomic composition of the predicted ISCST along with the hierarchical clustering dendrogram based on the relative abundance table that was used to predict the ISCSTs.

Following the species‐profiling analysis, in order to obtain a statistic‐based clustering of the samples based on their taxonomic composition, we performed the hierarchical cluster analysis (Difference between K Means and Hierarchical Clustering ‐ GeeksforGeeks, n.d.), leading to the generation of 20 clusters named from ISCSTs_1 to ISCSTs_20 with a clear and well‐defined microbiological profile (Fig. 2; Table S6). Notably, the high number of predicted clusters compared to adult microbiota meta‐analyses (Derrien *et al*., 2019) is compatible with the higher degree of interindividual variability during gut microbiota development (Milani *et al*., 2017; Derrien *et al*., 2019).

Intriguingly, the three most prevalent clusters were ISCSTs_8, ISCSTs_1 and ISCSTs_6. In detail, 317 samples (28.58% of total) fall into the ISCSTs_8, showing a bacterial community profile with *B*. *longum* as dominant taxa (average abundance of 41.78%) (Table S6; Fig. 2). In contrast, 172 samples (15.51% of total samples) were included in ISCSTs_1, showing a co‐dominance of *B*. *breve* (23.79% average abundance) and species belonging to the genus *Prevotella*, mainly *Prevotella copri* (9.02% average abundance) (Table S6; Fig. 2). Furthermore, ISCSTs_6, including 119 samples (10.73% of total samples), appeared to be the third most representative cluster, displaying a profile dominated mainly by the *Escherichia coli* species (average abundance of 40.72%) (Table S6; Fig. 2). Together, these results reinforced the notion that infants' gut microbiota is generally rich in the *Bifidobacterium* genus, followed by *E*. *coli* as reported in previous literature describing the pivotal role of these early gut colonizers in healthy infants (Vemuri *et al*., 2018).

Indeed, among the dominant taxa of the other relatively minor ISCSTs we still found various species of bifidobacteria, such as *B*. *bifidum* in ISCSTs_2 and *Bifidobacterium pseudocatenulatum* in Cluster_9, as well as multiple species belonging to the *Bacteroides* genera, such as *Bacteroides fragilis* in ISCSTs_15 and *Bacteroides uniformis* in ISCSTs_18 (Fig. 2; Table S2).

Furthermore, analysis of beta‐diversity depicted through 3D Principal Components analysis and correlation coefficient showed differences in the gut microbiota composition between the four age‐based groups (PERMANOVA *p*‐value <0.05) (Table S7; Fig. S3). This finding, coupled with those obtained from the alpha‐diversity analysis, corroborates with the notion that the infants' gut microbiota experiences rapid changes during the first year of life (Factors Influencing the Gut Microbiome in Children: From Infancy to Childhood ‐ PubMed, n.d.).

In this context, the 11 Côte d'Ivoire infants' samples, with age ranging from 1 to 90 days, enrolled in this study's framework showed average biodiversity of 14.54 species per sample, matching, as for age category 1–3 M.

Data regarding infant's age were also correlated to the 20 predicted clusters. Taking into account the age‐based samples subdivision, we found that ISCSTs_16, ISCSTs_17 and ISCSTs_18 constituted primarily (between 55.77% and 82.15% average abundance) by older infants (6–9 M and 9–12 M categories), which were associated with higher alpha diversity (*p*‐value <0.05) (Table S7), corresponding to an average of 23.9, 27.7 and 24.1 species per sample respectively (Figs S4 and S5). In contrast, ISCSTs_1, ISCSTs_2 and ISCSTs_6, formed primarily (between 79.07% and 92.31% average abundance) by infants below 6 months of age (1–3 M and 3–6 M categories), showed an average of 16, 12.3, and 12.8 species per sample respectively. These findings are consistent with previous literature, demonstrating the progressive increase in gut microbiota biodiversity with infant' age (Milani *et al*., 2017; Derrien *et al*., 2019).

## Species‐level comparison of Côte d'Ivoire infant against ISCSTs clusters

The large pool of 1098 datasets from different continents allowed us to compare the worldwide taxonomic variability of the gut microbiota of newborns during the first year of life with that of 11 newborns living in the Côte d'Ivoire. In detail, the taxonomic profile of each of the 11 Côte d'Ivoire infants was compared to the average profile observed for each ISCST. Moreover, it was also compared with the average composition identified for the 275 samples available in public databases corresponding to infants living in rural regions of Africa to identify key microbial signatures of gut microbiota development in pre‐urbanized countries.

**Fig. 3:**
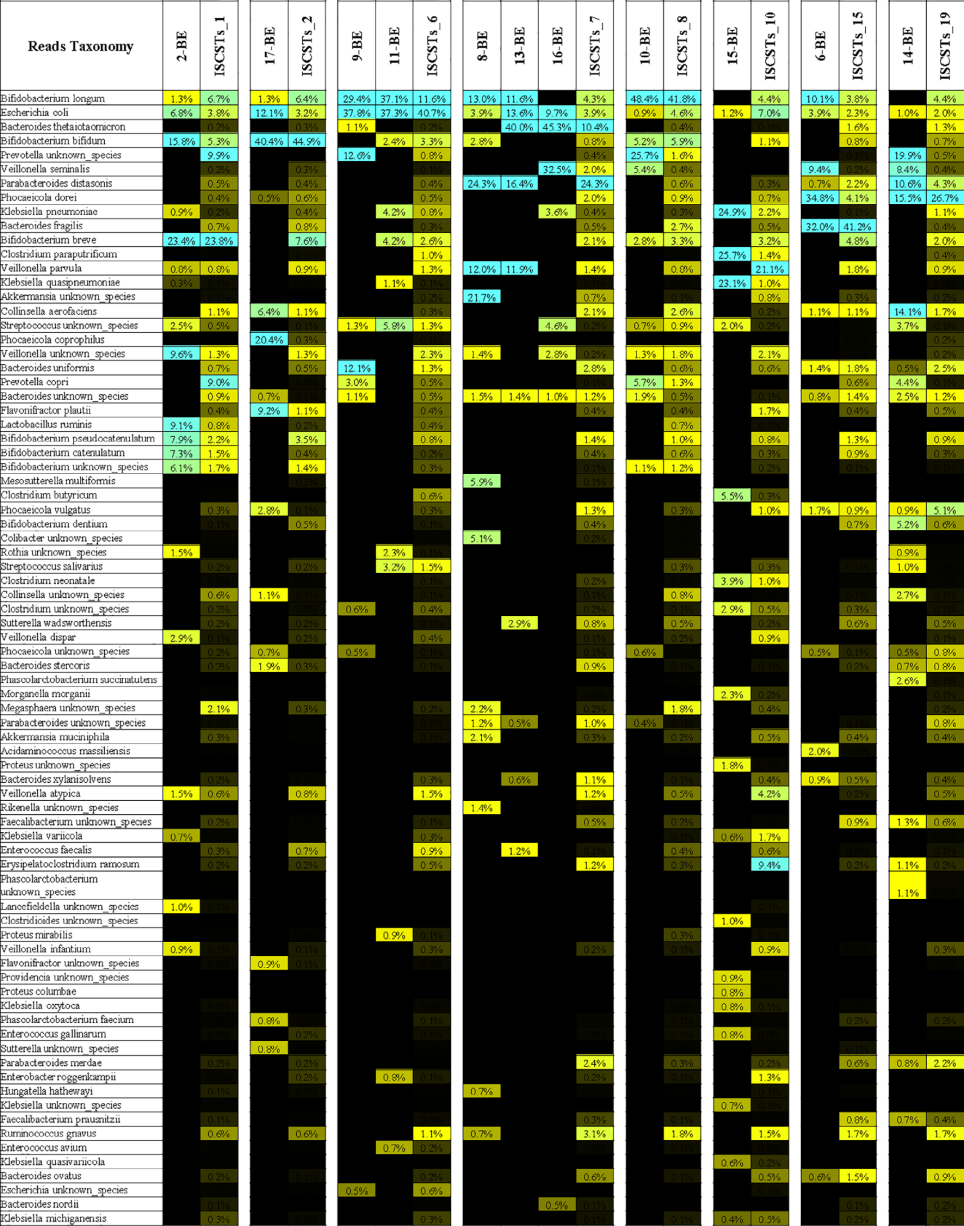
Comparison between 11 Côte d'Ivoire samples and ISCSTs. A colour‐graduated heatmap is reported in order to compare Côte d'Ivoire samples with predicted ISCSTs. Only taxa present in sub‐Saharan pool samples are graphically reported.

Intriguingly, 2‐BE, 17‐BE, 9‐BE, 8‐BE, 13‐BE, 16‐BE, 10‐BE and 11‐BE, fall in ISCSTs represented primarily by samples of age categories A and B (pre‐weaning), dominated by the bifidobacterial species such as *B*. *longum*, *B*. *bifidum*, *B*. *breve* and/or *E*. *coli*, i.e. ISCSTs 1, 2, 6, 7 and 8 (Figs 2 and 3). These data were in line with the age of the infants from which the samples were taken, which belonging to the 1–3 M category (<90 days of life) (Table S1; Fig. 2).

In contrast, sample 6‐BE was classified as a member of ISCST_15 due to the high relative abundance of *Bacteroides fragilis*. Remarkably, 28.83% of ISCST_15 is constituted by datasets of individuals of age category 9–12 M (>270 days), i.e. post‐weaning, as expected due to high relative abundance of *Bacteroides* species. These data may indicate the habit, in developing contexts, of introducing solid food early in the diet, especially concerning 6‐BE, a mixed feeding infant (Table S1).

The remaining samples 14‐BE and 15‐BE showed a high relative abundance of *Phocaeicola dorei* and *C*. *paraputrificum* along with various species of *Klebsiella* respectively, and were assigned to ISCST_19 and ISCST_10, (Figs 2 and 3; Table S6). Intriguingly, *C*. *paraputrificum* has been associated with paediatric infection, bacteremia, adult sepsis, and could indicate a risk for gut infections for 14‐BE and 15‐BE (Kiu *et al*., 2017; Intra *et al*., 2020).

The 11 samples of infants living in semi‐urban African context sequenced in this study were also compared to a dataset of 275 infants living in Africa with limited interaction with urbanized populations that were released in the framework of previous studies (Table S1) (Bender *et al*., 2016; Agapova *et al*., 2018). The latter datasets span from 1–3 M to 3–6 M age and are distributed mainly in three clusters associated with the same age range, i.e. ISCST_1, 6 and 8, due to dominance of three main taxa, *B*. *longum*, *E*. *coli* and *B*. *breve* (Table 1; Fig. 2). Notably, only 3.64% of the samples constituting this dataset fall in ISCSTs_2, 7 and 10, which are also associated with age groups 1–3 M/3–6 M and are highly represented in urbanized populations (17.89% of samples). This limited diversity in terms of ISCSTs observed for pre‐urbanized infants is probably correlated with a dietary intake in rural communities that causes the selection of a limited number of dominant species, especially compared to the much more varied diets of the urban infants (Voreades *et al*., 2014; Savage *et al*., 2018). Diet has been observed to influence the human milk oligosaccharides (HMO) profiles of lactating mothers. Thus, putatively affecting the selection of bacterial taxa in newborns by modulating the HMO profiles (Seferovic *et al*., 2020).

**Table 1:** Urban, rural and sub‐Saharan samples correlation with ISCSTs.

	Urban		Rural		11 Sub‐urban	
ISCSTs	Number of samples	%	Number of samples	%	Number of samples	%
1	105	12.76	66	24.00	1	9.09
2	61	7.41	3	1.09	1	9.09
3	15	1.82	0	0.00	0	0.00
4	7	0.85	1	0.36	0	0.00
5	11	1.34	0	0.00	0	0.00
6	73	8.87	44	16.00	2	18.18
7	37	4.50	3	1.09	3	27.27
8	173	21.02	143	52.00	1	9.09
9	19	2.31	6	2.18	0	0.00
10	50	6.08	4	1.45	1	9.09
11	36	4.37	2	0.73	0	0.00
12	11	1.34	0	0.00	0	0.00
13	14	1.70	0	0.00	0	0.00
14	12	1.46	1	0.36	0	0.00
15	45	5.47	2	0.73	1	9.09
16	28	3.40	0	0.00	0	0.00
17	19	2.31	0	0.00	0	0.00
18	52	6.32	0	0.00	0	0.00
19	38	4.62	0	0.00	1	9.09
20	17	2.07	0	0.00	0	0.00
Total samples	823		275		11	

Notably, in contrast to the 11 infant faecal samples from a semi‐urban area in Côte d'Ivoire, the 275 samples from two African rural communities showed a higher relative abundance of *B*. *longum* (*t*‐test *p*‐value <0.001) (Table S7).

## Covariance network analysis based on compositions of 1109 infants' samples

**Fig. 4:**
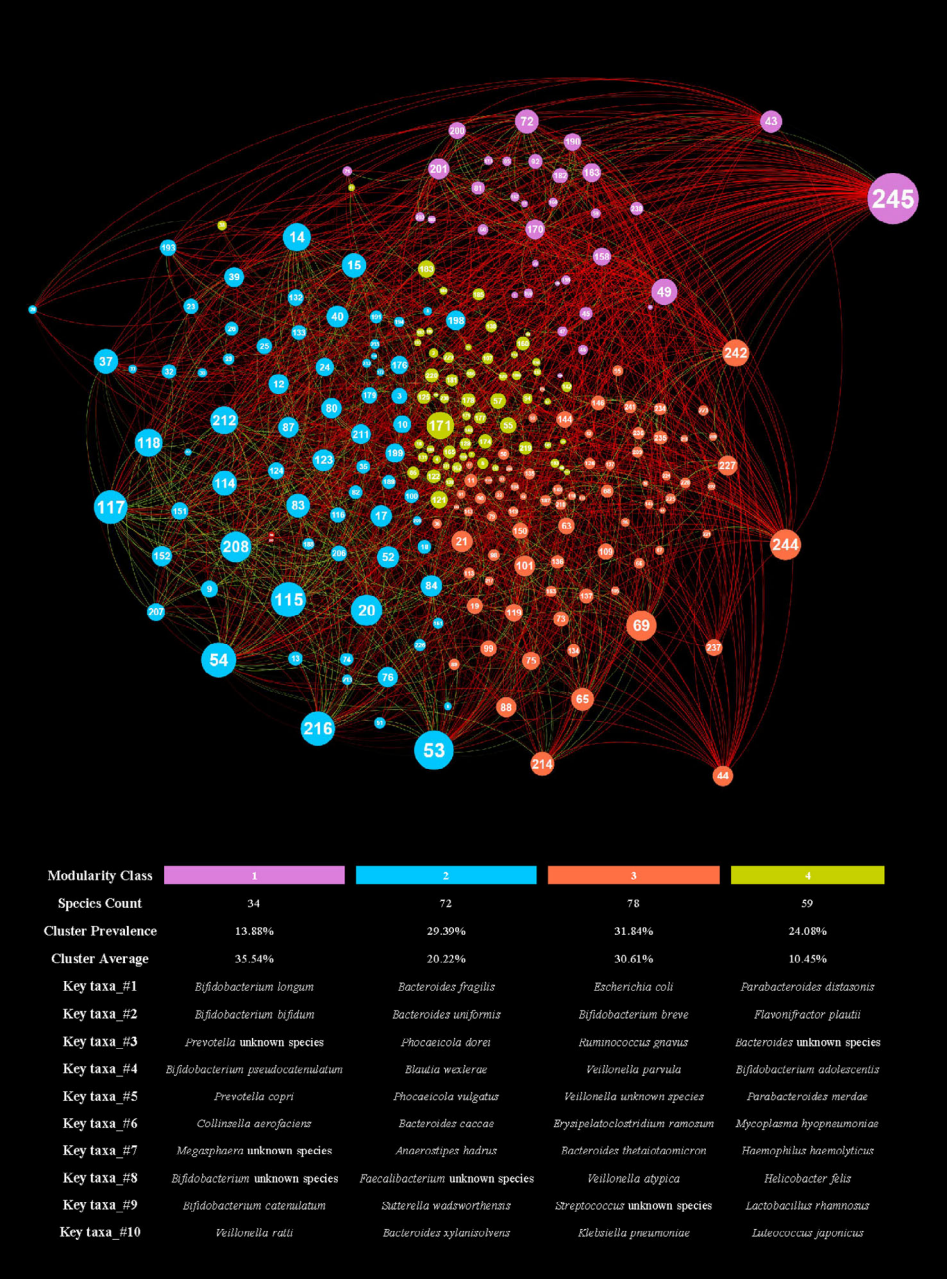
Network covariance of taxa observed in the 1109 samples included in the meta‐analysis. A network generated via Gephi software and force atlas 2 algorithm is reported in order to graphically represent the covariance relationship between each taxa observed in at least 10 samples. This filtering was made to remove background noise and enhance the clearness of the image.

To define the intricate positive and negative relationships existing between the bacterial species constituting the 1109 infants' faecal samples, we performed a bivariate covariance analysis based on the Pearson correlations with a bootstrap value of 1000 repetitions (Table S8) (Software SPSS ‐ Italia | IBM, n.d.). To remove background noise, we selected the bacterial species present in more than 10 samples. The data resulting from the covariance analysis were exploited to create an interaction network with Gephy software (Gephi ‐ The Open Graph Viz Platform, n.d.) with a modularity value set to 1.5 (Fig. 4; Table S9) (Brandes *et al*., 2008). This analysis allowed us to identify four Modularity Cluster (MC), named MC_1, MC_2, MC_3 and MC_4, in which the species forming a given cluster tend to correlate positively with each other and negatively with the members of other clusters.

In detail, MC_1 is mainly constituted by health‐promoting taxa typically associated with the early gut microbiota development, such as members of the *Prevotella* and *Bifidobacterium* genera, including *B*. *longum*, *B*. *bifidum* and *P*. *copri*. Interestingly, although a limited range of microbial taxa encompassed this cluster (13.88% of the analysed species), they corresponded to a sum of 35.54% of average abundance (Fig. 4; Table S9). Moreover, MC_2 is mainly composed of species belonging to *Bacteroides*, *Phocaeicola* and *Faecalibacterium* genera, such as *Bacteroides fragilis*, *P*. *dorei* and *Faecalibacterium prausnitzii*, which have been previously correlated with a healthy adult gut microbiota environment. This cluster covers 29.39% of all the analysed species, corresponding to a total summed average abundance of 20.22% (Fig. 3; Table S9). Intriguingly, these two clusters highlight that a specific set of bacterial taxa seem to cooperate for the subsequent colonization of the two peculiar ecological niches represented by the gut environment of pre‐weaning (MC_1) and post‐weaning (MC_2) infants.

Furthermore, MC_3 is composed primarily of species belonging to *Escherichia*, *Veillonella*, *Klebsiella* and *Clostridium* genera, covering 31.84% of all the analysed species, corresponding to a sum of an average abundance of 30.61% (Fig. 4; Table S9). This cluster highlighted how potentially health‐threatening species, such as *E*. *coli* and *Clostridium*, tend to correlate positively with each other. However, this does not mean that they can directly cause harm to the host's health (Christofi *et al*., 2019). Members of these taxa are commonly found in the healthy intestinal microbiota and are also engaged in positive interactions with probiotic species such as *B*. *breve* (Cukrowska *et al*., 2020). Remarkably, the latter result supports the notion that species belonging to *Escherichia*, *Veillonella*, *Klebsiella* and *Clostridium* genera may act as opportunistic pathogens, i.e. may negatively affect the host's health only in specific conditions of altered gut microbiota homeostasis.

Lastly, MC_4 is a cluster composed primarily of accessory taxa, including *Parabacteroides distasonis*, *Flavonifractor plautii* and *Bifidobacterium adolescentis*, which showed weak positive interactions with the other dominant clusters. This cluster covers 24.08% of all analysed species, corresponding to a sum of average abundances of only 10.45% (Fig. 4; Table S9). Notably, this cluster encompassed a wide range of bacterial species typically found across all ages as minor players of the gut community. Thus, suggesting that these bacterial taxa, despite their possible cooperation for efficient niche colonization, tend to limitedly interact with members of other MC clusters and may exert a key role in defining inter‐individual variability in the gut microbiota composition (Almeida *et al*., 2019; Yang *et al*., 2020).

By correlating the compositions of the 11 Côte d'Ivoire infants with the MCs, we observed that most of their taxonomic composition falls into MC_3 and MC_1 apart from the 6‐BE sample, which has >70% of its taxonomic composition belonging to MC_2 (Fig. S6; Table S9). Intriguingly, only 15‐BE and 16‐BE have >90% of their taxonomic composition belonging to MC_3, while showing limited participation of MC_1. This could indicate a possible onset of future pathogenic dysbiotic states due to the marked dominance of opportunistic pathogens (Fig. S6; Table S9).

Furthermore, by correlating rural, semi‐urban and urban samples with MCs, we revealed that in rural datasets the species belonging to MC_1 make up for 66.26% of the total taxonomic composition, while MC_1 covers only 25.31% in urban datasets. In rural datasets, an additional 25.16% of the total taxonomic composition belongs to MC_3. At the same time, urban samples are characterized by higher variability, with 25.64% of the taxonomic composition belonging to MC_2, 32.23% to the MC_3 cluster and 12.96% to MC_4 (Fig. S6).

## Conclusions

The large pool of shotgun metagenomics datasets corresponding to 1098 infants included in this meta‐analysis allowed us to explore the worldwide taxonomic variability of the infant gut microbiota across the first year of life. Furthermore, in the framework of this study, we collected a faecal sample from 11 infants living in Côte d'Ivoire. The latter, along with two additional publicly available datasets corresponding to infants living in pre‐urbanized populations, revealed microbial signatures associated with pre‐urbanization. Intriguingly, data collected suggest that the geographical origin and diet of pre‐urbanized populations impact the overall microbial biodiversity of the infant gut microbiota. In fact, mothers diet in pre‐urbanized areas, defined by high consumption of simple sugars, seasonal foods and fibre intake, can indirectly determine differences in infant's gut microbiota through the modulation of HMO's profiles of lactating mothers (Seferovic *et al*., 2020). Indeed, modulation of the prevalence of the main enterotypes, i.e. ISCSTs, was predicted by statistical integration of all the taxonomic profiles retrieved by the meta‐analysis. Remarkably, while this study revealed intriguing data, further investigations with additional larger cohorts are needed to validate and extend our knowledge of the gut microbiota development of infants living in pre‐urbanized areas.

## Author Contributions

R.D.A. conceived the study, performed sample collections and wrote the manuscript. F.F. performed bioinformatic analyses and wrote the manuscript. C.M. performed bioinformatic analyses, conceived the study and wrote the manuscript. L.M., G.A.L. and C.T. performed data analysis. R.A., G.L., G.A. and A.V. performed microbial DNA sequencing. M.V. and F.T. revised and approved the manuscript. R.F., M.B. and K.H.M. performed sample collections. All authors reviewed the manuscript.

## Supporting information


**Appendix S1:** Supplementary File.

**Table S1.** Metadata of samples included in this meta‐analysis.
**Table S2**. 16S rRNA sequencing‐based taxonomic profiling of the 11 Côte d'Ivoire samples.
**Table S3**. Shallow shotgun‐based taxonomic profiling of the 11 Côte d'Ivoire samples.
**Table S4**. Shallow shotgun‐based taxonomic profiling of the 1109 samples included in this meta‐analysis.
**Table S5**. Full heatmap presence‐absence of all the bacterial species detected in the 1109 samples included in this meta‐analysis.
**Table S6**. Average ISCSTs taxonomic composition at species‐level.
**Table S7**. Table of all the statistical analyses performed in this meta‐analysis.
**Table S8**. Pearson‐based matrix of the covariances observed between bacterial taxa predicted in at least 10 samples included in the meta‐analysis.
**Table S9**. Taxonomic composition of the predicted modularity clusters.

**Fig. S1.** Metadata of the 1109 samples included in this meta‐analysis. In panel a is reported a cake graph explaining geographic subdivision of the 1109 samples. In panel b is reported a cake graph showing age subdivision of the 1109 samples.
**Fig. S2.** Graphic comparison between 16S rRNA gene microbial profiling and shallow metagenomic profiling at genus level. In panel a a bar plot is reported in order to show average abundance compositions at genera level retrieved through shallow shotgun profiling. In panel b a bar plot is displayed in order to show average abundance compositions at genera level obtained by 16S rRNA gene microbial profiling, and only taxa >0.1% Average are showed for cleanness.
**Fig. S3.** Beta‐diversity analysis of the 1109 samples included in the meta‐analysis. Panels a and b show a PCoA representation based on the Bray‐Curtis index and the species‐level taxonomic profile obtained for the 1109 samples included in the meta‐analysis. The samples are coloured based on age groups in panel a and based on ISCST in panel b.
**Fig. S4.** ISCSTs age compositions. In panel a is shown a bar plot representation of the age group composition of every ISCSTs as sample counts. In panel b is reported a bar plot representation of the age group composition of every ISCSTs as percentage of the whole ISCST. Panel c provides a detailed summary of all the metadata associated with the predicted ISCSTs
**Fig. S5.** Average alpha diversity of the predicted ISCSTs. In panel a is reported a bar plot representation of the raw count of the number of species identified in each ISCSTs. Panel b shows a bar plot representing the average number of species (Alpha diversity) correlated to each ISCSTs.
**Fig. S6.** Modularity clusters correlated to the 11 sub‐Saharan samples. In panel a is reported a bar plot representation of sub‐Saharan sample composition in terms of previously defined MCs. Panel b shows a table detailed data regarding composition in terms of previously predicted MCs.

## Data Availability

The SRA accession number of the metagenomic sequences of the 11 Côte d'Ivoire infant faecal sample sequenced in this study is SRP311268.

## References

[emi412960-bib-0001] Adak, A. , and Khan, M.R. (2019) An insight into gut microbiota and its functionalities. Cell Mol Life Sci 76: 473–493. 10.1007/s00018-018-2943-4. 30317530 PMC11105460

[emi412960-bib-0002] Agapova, S.E. , Stephenson, K.B. , Divala, O. , Kaimila, Y. , Maleta, K.M. , Chrissie, T. , *et al*. (2018) Additional common bean in the diet of Malawian children does not affect linear growth, but reduces intestinal permeability. J Nutr 148: 267–274. 10.1093/jn/nxx013. 29490090

[emi412960-bib-0003] Almeida, A. , Mitchell, A.L. , Boland, M. , Forster, S.C. , Gloor, G.B. , Tarkowska, A. , *et al*. (2019) A new genomic blueprint of the human gut microbiota. Nature 568: 499–504. 10.1038/s41586-019-0965-1. 30745586 PMC6784870

[emi412960-bib-0004] Bender, J.M. , Li, F. , Martelly, S. , Byrt, E. , Rouzier, V. , Leo, M. , *et al*. (2016) Maternal HIV infection influences the microbiome of HIV‐uninfected infants. Sci Transl Med 8: 349ra100. 10.1126/scitranslmed.aaf5103. PMC5301310 27464748

[emi412960-bib-0005] Brandes, U. , Delling, D. , Gaertler, M. , Görke, R. , Hoefer, M. , Nikoloski, Z. , and Wagner, D. (2008) On modularity clustering. IEEE Trans Knowl Data Eng 20: 172–188.

[emi412960-bib-0006] Christofi, T. , Panayidou, S. , Dieronitou, I. , Michael, C. , and Apidianakis, Y. (2019) Metabolic output defines *Escherichia coli* as a health‐promoting microbe against intestinal *Pseudomonas aeruginosa* . Sci Rep 9: 14463. 10.1038/s41598-019-51058-3. 31595010 PMC6783455

[emi412960-bib-0007] Costea, P.I. , Hildebrand, F. , Manimozhiyan, A. , Bäckhed, F. , Blaser, M.J. , Bushman, F.D. , *et al*. (2017) Enterotypes in the landscape of gut microbial community composition. Nat Microbiol 3: 8–16. 10.1038/s41564-017-0072-8. 29255284 PMC5832044

[emi412960-bib-0008] Cukrowska, B. , Bierła, J.B. , Zakrzewska, M. , Klukowski, M. , and Maciorkowska, E. (2020) The relationship between the infant gut microbiota and allergy. The role of Bifidobacterium breve and prebiotic oligosaccharides in the activation of anti‐allergic mechanisms in early life. Nutrients 12: 946. 10.3390/nu12040946. PMC7230322 32235348

[emi412960-bib-0009] Decuypere, S. , Meehan, C.J. , Van Puyvelde, S. , De Block, T. , Maltha, J. , Palpouguini, L. , *et al*. (2016) Diagnosis of bacterial bloodstream infections: a 16S metagenomics approach. PLoS Negl Trop Dis 10: e0005811. 10.1371/journal.pntd.0004470. PMC4771206 26927306

[emi412960-bib-0010] Derrien, M. , Alvarez, A.S. , and de Vos, W.M. (2019) The gut microbiota in the first decade of life. Trends Microbiol 27: 997–1010. 10.1016/j.tim.2019.08.001. 31474424

[emi412960-bib-0011] Difference between K Means and Hierarchical Clustering ‐ GeeksforGeeks . n.d. URL https://www.geeksforgeeks.org/difference-between-k-means-and-hierarchical-clustering/.

[emi412960-bib-0012] Le Doare, K. , Holder, B. , Bassett, A. , and Pannaraj, P.S. (2018) Mother's milk: a purposeful contribution to the development of the infant microbiota and immunity. Front Immunol 9: 361. 10.3389/fimmu.2018.00361. 29599768 PMC5863526

[emi412960-bib-0013] Factors Influencing the Gut Microbiome in Children: From Infancy to Childhood ‐ PubMed . n.d. URL https://pubmed.ncbi.nlm.nih.gov/31180062/. 31180062

[emi412960-bib-0014] Gentile, C.L. , and Weir, T.L. (2018) The gut microbiota at the intersection of diet and human health. Science 362: 776–780. 10.1126/science.aau5812. 30442802 PMC13264711

[emi412960-bib-0015] Gephi ‐ The Open Graph Viz Platform . n.d. URL https://gephi.org/.

[emi412960-bib-0016] Hillmann, B. , Al‐Ghalith, G.A. , Shields‐Cutler, R.R. , Zhu, Q. , Gohl, D.M. , Beckman, K.B. , *et al*. (2018) Evaluating the information content of shallow shotgun metagenomics. MSystems 3: e00069–18. 10.1128/msystems.00069-18. 30443602 PMC6234283

[emi412960-bib-0017] Intra, J. , Milano, A. , Sarto, C. , and Brambilla, P. (2020) A rare case of clostridium Paraputrificum bacteremia in a 78‐year‐old Caucasian man diagnosed with an intestinal neoplasm. Anaerobe 66: 102292. 10.1016/j.anaerobe.2020.102292. 33171286

[emi412960-bib-0018] Kiu, R. , Caim, S. , Alcon‐Giner, C. , Belteki, G. , Clarke, P. , Pickard, D. , *et al*. (2017) Preterm infant‐associated clostridium Tertium, clostridium Cadaveris, and clostridium Paraputrificum strains: genomic and evolutionary insights. Genome Biol Evol 9: 2707–2714. 10.1093/gbe/evx210. 29044436 PMC5647805

[emi412960-bib-0019] Laudadio, I. , Fulci, V. , Palone, F. , Stronati, L. , Cucchiara, S. , and Carissimi, C. (2018) Quantitative assessment of shotgun metagenomics and 16S RDNA amplicon sequencing in the study of human gut microbiome. OMICS 22: 248–254. 10.1089/omi.2018.0013. 29652573

[emi412960-bib-0020] De Leoz, M.L.A. , Kalanetra, K.M. , Bokulich, N.A. , Strum, J.S. , Underwood, M.A. , German, J.B. , *et al*. (2015) Human Milk Glycomics and gut microbial genomics in infant feces show a correlation between human Milk oligosaccharides and gut microbiota: a proof‐of‐concept study. J Proteome Res 14: 491–502. 10.1021/pr500759e. 25300177 PMC4286166

[emi412960-bib-0021] Lugli, G.A. , Duranti, S. , Milani, C. , Mancabelli, L. , Turroni, F. , Alessandri, G. , *et al*. (2020) Investigating Bifidobacteria and human Milk oligosaccharide composition of lactating mothers. FEMS Microbiol Ecol 96: fiaa049. 10.1093/femsec/fiaa049. 32188978

[emi412960-bib-0022] Mancabelli, L. , Tarracchini, C. , Milani, C. , Lugli, G.A. , Fontana, F. , Turroni, F. , *et al*. (2020) Multi‐population cohort meta‐analysis of human intestinal microbiota in early life reveals the existence of infant community state types (ICSTs). Comput Struct Biotechnol J 18: 2480–2493. 10.1016/j.csbj.2020.08.028. 33005310 PMC7516180

[emi412960-bib-0023] Marchesi, J.R. , Adams, D.H. , Fava, F. , Hermes, G.D.A. , Hirschfield, G.M. , Hold, G. , *et al*. (2016) The gut microbiota and host health: a new clinical frontier. Gut 65: 330–339. 10.1136/gutjnl-2015-309990. 26338727 PMC4752653

[emi412960-bib-0024] Matamoros, S. , Gras‐Leguen, C. , Le Vacon, F. , Potel, G. , and De La Cochetiere, M.F. (2013) Development of intestinal microbiota in infants and its impact on health. Trends Microbiol 21: 167–173. 10.1016/j.tim.2012.12.001. 23332725

[emi412960-bib-0025] Milani, C. , Casey, E. , Lugli, G.A. , Moore, R. , Kaczorowska, J. , Feehily, C. , *et al*. (2018) Tracing mother‐infant transmission of bacteriophages by means of a novel analytical tool for shotgun metagenomic datasets: METAnnotatorX. Microbiome 6: 145. 10.1186/s40168-018-0527-z. 30126456 PMC6102903

[emi412960-bib-0026] Milani, C. , Duranti, S. , Bottacini, F. , Casey, E. , Turroni, F. , Mahony, J. , *et al*. (2017) The first microbial colonizers of the human gut: composition, activities, and health implications of the infant gut microbiota. Microbiol Mol Biol Rev 81: e00036‐17. 10.1128/mmbr.00036-17. PMC5706746 29118049

[emi412960-bib-0027] Nagpal, R. , and Yamashiro, Y. (2018) Gut microbiota composition in healthy Japanese infants and young adults born by C‐section. Ann Nutr Metab 73: 4–11. 10.1159/000490841. 30041174

[emi412960-bib-0028] Rutayisire, E. , Huang, K. , Liu, Y. , and Tao, F. (2016) The mode of delivery affects the diversity and colonization pattern of the gut microbiota during the first year of Infants' life: a systematic review. BMC Gastroenterol 16: 86. 10.1186/s12876-016-0498-0. 27475754 PMC4967522

[emi412960-bib-0029] Savage, J.H. , Lee‐Sarwar, K.A. , Sordillo, J.E. , Lange, N.E. , Zhou, Y. , O'Connor, G.T. , *et al*. (2018) Diet during pregnancy and infancy and the infant intestinal microbiome. J Pediatr 203: 47–54.e4. 10.1016/j.jpeds.2018.07.066. 30173873 PMC6371799

[emi412960-bib-0030] Savino, F. , Cresi, F. , Pautasso, S. , Palumeri, E. , Tullio, V. , Roana, J. , *et al*. (2004) Intestinal microflora in breastfed colicky and non‐colicky infants. Acta Paediatr 93: 825–829. 10.1111/j.1651-2227.2004.tb03025.x. 15244234

[emi412960-bib-0031] Schirbel, A. , and Fiocchi, C. (2011) Targeting the innate immune system in pediatric inflammatory bowel disease. Expert Rev Gastroenterol Hepatol 5: 33–41. 10.1586/egh.10.76. 21309670

[emi412960-bib-0032] Seferovic, M.D. , Mohammad, M. , Pace, R.M. , Engevik, M. , Versalovic, J. , Bode, L. , *et al*. (2020) Maternal diet alters human Milk oligosaccharide composition with implications for the Milk metagenome. Sci Rep 10: 22092. 10.1038/s41598-020-79022-6. 33328537 PMC7745035

[emi412960-bib-0033] Software SPSS ‐ Italia | IBM . n.d. URL https://www.ibm.com/it-it/analytics/spss-statistics-software.

[emi412960-bib-0034] Turroni, F. , Milani, C. , Duranti, S. , Lugli, G.A. , Bernasconi, S. , Margolles, A. , *et al*. (2020) The infant gut microbiome as a microbial organ influencing host well‐being. Italian J Pediatr 46: 16. 10.1186/s13052-020-0781-0. PMC7003403 32024556

[emi412960-bib-0035] Vemuri, R. , Gundamaraju, R. , Shastri, M.D. , Shukla, S.D. , Kalpurath, K. , Ball, M. , *et al*. (2018) Gut microbial changes, interactions, and their implications on human lifecycle: an ageing perspective. BioMed Res Int 2018: 4178607. 10.1155/2018/4178607. 29682542 PMC5846367

[emi412960-bib-0036] Voreades, N. , Kozil, A. , and Weir, T.L. (2014) Diet and the development of the human intestinal microbiome. Front Microbiol 5: 494. 10.3389/fmicb.2014.00494. 25295033 PMC4170138

[emi412960-bib-0037] Yadav, M. , Verma, M.K. , and Chauhan, N.S. (2018) A review of metabolic potential of human gut microbiome in human nutrition. Arch Microbiol 200: 203–217. 10.1007/s00203-017-1459-x. 29188341

[emi412960-bib-0038] Yang, J. , Ji, P. , Lu, S. , Bai, X. , Wu, Y. , Dong, J. , *et al*. (2020) Species‐level analysis of human gut microbiota with Metataxonomics. Front Microbiol 11: 2029. 10.3389/FMICB.2020.02029. 32983030 PMC7479098

