# Supplementary material for: Investigating the infant gut microbiota in developing countries: worldwide metagenomic meta‐analysis involving infants living in sub‐urban areas of Côte d'Ivoire
Source: Environ Microbiol Rep. 2021 Jun 21;13(5):626–36. doi: 10.1111/1758-2229.12960 (PMC8518733; doi:10.1111/1758-2229.12960)
Supplement: Supplementary file 1 — Appendix S1: Supplementary File. [file EMI4-13-626-s003.docx]

**Investigating the infant gut microbiota in developing countries: worldwide metagenomic meta-analysis involving infants living in sub-urban areas of Côte d’Ivoire**

Keywords: Sub-Saharan Africa, Enterotypes, Community State Types, Diet

Federico Fontana^1,2^, Leonardo Mancabelli^1^, Gabriele Andrea Lugli^1^, Chiara Taracchini^1^, Giulia Alessandri^1^, Giulia Longhi^1,2^, Rosaria Anzalone^2^, Alice Viappiani^2^, Roch Famo^4^, Marc Brognan^4^, Kouamé Hervé Micondo^4^, Francesca Turroni^1,3^, Marco Ventura^1,3^, Rossella D’Alfonso^4,5,6*^, and Christian Milani^1,3*^

Laboratory of Probiogenomics, Department of Chemistry, Life Sciences, and Environmental Sustainability, University of Parma, Parma, Italy^1^; GenProbio srl, Parma, Italy^2^; Microbiome Research Hub, University of Parma, Parma, Italy^3^; Centre Médical Don Orione Anyama, Anyama, Côte d’Ivoire^4^; Pediatric Service of Hospital Military D'Abidjan, Abidjan, Côte d’Ivoire^5^; Department of Systems Medicine, University of Rome Tor Vergata, Rome, Italy^6^

* These authors contributed equally

Correspondence. Mailing address for Christian Milani Laboratory of Probiogenomics, Department of Chemistry, Life Sciences, and Environmental Sustainability, University of Parma, Parco Area delle Scienze 11a, 43124 Parma, Italy. Phone: ++39-0521-904785. E-mail: christian.milani@unipr.it

Mailing address for Rossella D’Alfonso, Department of Systems Medicine, University of Rome Tor Vergata, Via Cracovia, 50, 00133, Rome, Italy. Phone: ++39-06-72596894. E-mail: dalfonso@uniroma2.it

**Experimental Procedures**

**Recruitment**

11 infants of Sub-Saharan geographical origin were enrolled in this study. The intent was to generate preliminary data from a small set of patients from a specific geographical origin to compare to the largest dataset of infants of different and quite-similar geographical origins.

The study protocol was approved by the Independent Ethics Committee of Centre Médical Don Orione Anyama and by the Independent Ethics Committee of Fondazione Policlinico Tor Vergata. Written informed consent was obtained from parents of all recruited infants.

**Samples collection and DNA extraction**

The samples were self-collected in the morning by spontaneous evacuation and stored at room temperature in stool nucleic acid collection and preservation tubes (Norgen Biotek, Thorold, Niagara, Ontario, Canada) containing 2 mL of preservative and inactivating solution. Samples were then delivered by participants to the research center within 24 h and then refrigerated at room temperature.

For the purpose of this study, from a total of 11 samples of Sub-Sahara African infants, Bacterial DNA was extracted from fecal samples using the QIAamp Fast DNA Stool Mini kit following the manufacturer's instructions (Qiagen Ltd., Strasse, Germany) and quantified using fluorometric Qubit quantification system (Life Technologies, Thermo Fisher Scientific, Waltham, Massachusetts, USA).

**Database selections**

Datasets were retrieved by publicly available resources in NCBI. Subsequently, from each dataset only samples of infants with matching–criteria metadata (Healthy status, age < 1 year, No antibiotic intake) were finally retrieved, for a total of 1109 samples based on Illumina sequencing.

**16S rRNA sequencing and profiling**

To avoid biases caused by different bioinformatic analysis pipelines, the sequence read pools of each dataset were filtered and analyzed through the same custom script based on the QIIME 2 software suite. Quality control-maintained sequences with a length between 140 and 400 bp and average sequence quality score of >20, while sequences with homopolymers of >7 bp and mismatched primers were omitted. 16S rRNA Operational Taxonomic Units (OTUs) were defined at 100% sequence homology using DADA2 and OTUs that were represented by just a single sequence were removed. All reads were classified to the lowest possible taxonomic rank using QIIME 2,  and a reference dataset from the SILVA database v.132.

**Shallow shotgun sequencing**

A DNA library was prepared using the Nextera XT DNA sample preparation kit (Illumina, San Diego, California, USA) according to the manufacturer's instructions. In detail, one ng input DNA from each sample was used for library preparation. The isolated DNA underwent enzymatically fragmentation, adapter ligation, and purification involving magnetic beads.

Then, samples were quantified using a fluorometric Qubit quantification system (Life Technologies, Thermo Fisher Scientific, Waltham, Massachusetts, USA) loaded on a 2200 Tape Station Instrument (Agilent Technologies, Santa Clara, California, USA) and normalized to 4 nM. Sequencing was performed on a MiSeq instrument (Illumina, San Diego, California, USA), according to the manufacturer's instructions, using the 2 × 250 MiSeq Reagent Kit v3 (600-cycle), and spike-in of 1% PhiX control library.

**Shallow taxonomic profiling**

Taxonomic profiling of sequenced reads was performed with the METAnnotatorX bioinformatics platform (Computational Microbiology Unit, University of Parma, Parma, Italy) (Milani et al. 2018). In detail, the raw data in fastq format were submitted to quality filtering with removal of reads with an average quality <25. Subsequently, host DNA was removed by reads mapping to the human genome.

Retained sequences were used as input to perform a MegaBLAST local alignment of reads to pre-processed database including available genomes of eukaryotes (Fungi and Protists), bacteria, archaea, and viruses. Reads showing a nucleotide identity >94% to the genomes included in the database were classified at the species level, while if a lower percentage identity was detected, they were classified at the genus level as undefined species. These cut-offs are those generally employed for the ANI taxonomic assignment of genomes.

Functional profiling of sequenced reads was performed with the METAnnotatorX bioinformatics platform.

**Statistics and Cluster analysis**

HCL analysis was performed on OriginLabPro 2021 (“Origin 2021 Feature Highlights” n.d.).

PCoA Analysis and Permanova statistic were performed on Qiime2 (Bolyen et al. 2019) .

HCL was made with observations and cluster method of furthest neighbor with a distance Type of Pearson correlations. Clustroid is found by Sum of squares of distance.

PCoA was run based on a Correlation matrix, explaining 3 Principal Components, and excluding missing values through listwise approach.

**Supplementary Table Legend**

**Table S1. Metadata of samples included in this meta-analysis.**

**Table S2. 16S rRNA sequencing-based taxonomic profiling of the 11 Côte d’Ivoire samples.**

**Table S3. Shallow shotgun-based taxonomic profiling of the 11 Côte d’Ivoire samples.**

**Table S4. Shallow shotgun-based taxonomic profiling of the 1109 samples included in this meta-analysis.**

**Table S5. Full heatmap presence-absence of all the bacterial species detected in the 1109 samples included in this meta-analysis.**

**Table S6. Average ISCSTs taxonomic composition at species-level.**

**Table S7. Table of all the statistical analyses performed in this meta-analysis.**

**Table S8. Pearson-based matrix of the covariances observed between bacterial taxa predicted in at least 10 samples included in the meta-analysis.**

**Table S9. Taxonomic composition of the predicted modularity clusters.**

**Supplementary Figure Legends**

**Figure S1. Metadata of the 1109 samples included in this meta-analysis.** In panel a is reported a cake graph explaining geographic subdivision of the 1109 samples. In panel b is reported a cake graph showing age subdivision of the 1109 samples.

**Figure S2. Graphic comparison between 16S rRNA gene microbial profiling and shallow metagenomic profiling at genus level.** In panel a a bar plot is reported in order to show average abundance compositions at genera level retrieved through shallow shotgun profiling. In panel b a bar plot is displayed in order to show average abundance compositions at genera level obtained by 16S rRNA gene microbial profiling, and only taxa > 0.1% Average are showed for cleanness.

**Figure S3. Beta-diversity analysis of the 1109 samples included in the meta-analysis.** Panels a and b show a PCoA representation based on the Bray-Curtis index and the species-level taxonomic profile obtained for the 1109 samples included in the meta-analysis. The samples are colored based on age groups in panel a and based on ISCST in panel b.

**Figure S4. ISCSTs age compositions.** In panel a is shown a bar plot representation of the age group composition of every ISCSTs as sample counts. In panel b is reported a bar plot representation of the age group composition of every ISCSTs as percentage of the whole ISCST. Panel c provides a detailed summary of all the metadata associated with the predicted ISCSTs

**Figure S5. Average alpha diversity of the predicted ISCSTs.** In panel a is reported a bar plot representation of the raw count of the number of species identified in each ISCSTs. Panel b shows a bar plot representing the average number of species (Alpha diversity) correlated to each ISCSTs.

**Figure S6. Modularity clusters correlated to the 11 sub-saharan samples.** In panel a is reported a bar plot representation of sub-saharan sample composition in terms of previously defined MCs. Panel b shows a table detailed data regarding composition in terms of previously predicted MCs.
