# Supplementary material for: Investigating the infant gut microbiota in developing countries: worldwide metagenomic meta‐analysis involving infants living in sub‐urban areas of Côte d'Ivoire
Source: Environ Microbiol Rep. 2021 Jun 21;13(5):626–36. doi: 10.1111/1758-2229.12960 (PMC8518733; doi:10.1111/1758-2229.12960)
Supplement: Supplementary file 3 — Fig. S1. Metadata of the 1109 samples included in this meta‐analysis. In panel a is reported a cake graph explaining geographic subdivision of the 1109 samples. In panel b is reported a cake graph showing age subdivision of the 1109 samples. Fig. S2. Graphic comparison between 16S rRNA gene microbial profiling and shallow metagenomic profiling at genus level. In panel a a bar plot is reported in order to show average abundance compositions at genera level retrieved through shallow shotgun profiling. In panel b a bar plot is displayed in order to show average abundance compositions at genera level obtained by 16S rRNA gene microbial profiling, and only taxa >0.1% Average are showed for cleanness. Fig. S3. Beta‐diversity analysis of the 1109 samples included in the meta‐analysis. Panels a and b show a PCoA representation based on the Bray‐Curtis index and the species‐level taxonomic profile obtained for the 1109 samples included in the meta‐analysis. The samples are coloured based on age groups in panel a and based on ISCST in panel b. Fig. S4. ISCSTs age compositions. In panel a is shown a bar plot representation of the age group composition of every ISCSTs as sample counts. In panel b is reported a bar plot representation of the age group composition of every ISCSTs as percentage of the whole ISCST. Panel c provides a detailed summary of all the metadata associated with the predicted ISCSTs Fig. S5. Average alpha diversity of the predicted ISCSTs. In panel a is reported a bar plot representation of the raw count of the number of species identified in each ISCSTs. Panel b shows a bar plot representing the average number of species (Alpha diversity) correlated to each ISCSTs. Fig. S6. Modularity clusters correlated to the 11 sub‐Saharan samples. In panel a is reported a bar plot representation of sub‐Saharan sample composition in terms of previously defined MCs. Panel b shows a table detailed data regarding composition in terms of previously predicted MCs. [file EMI4-13-626-s002.pdf]

a)

Geographic location

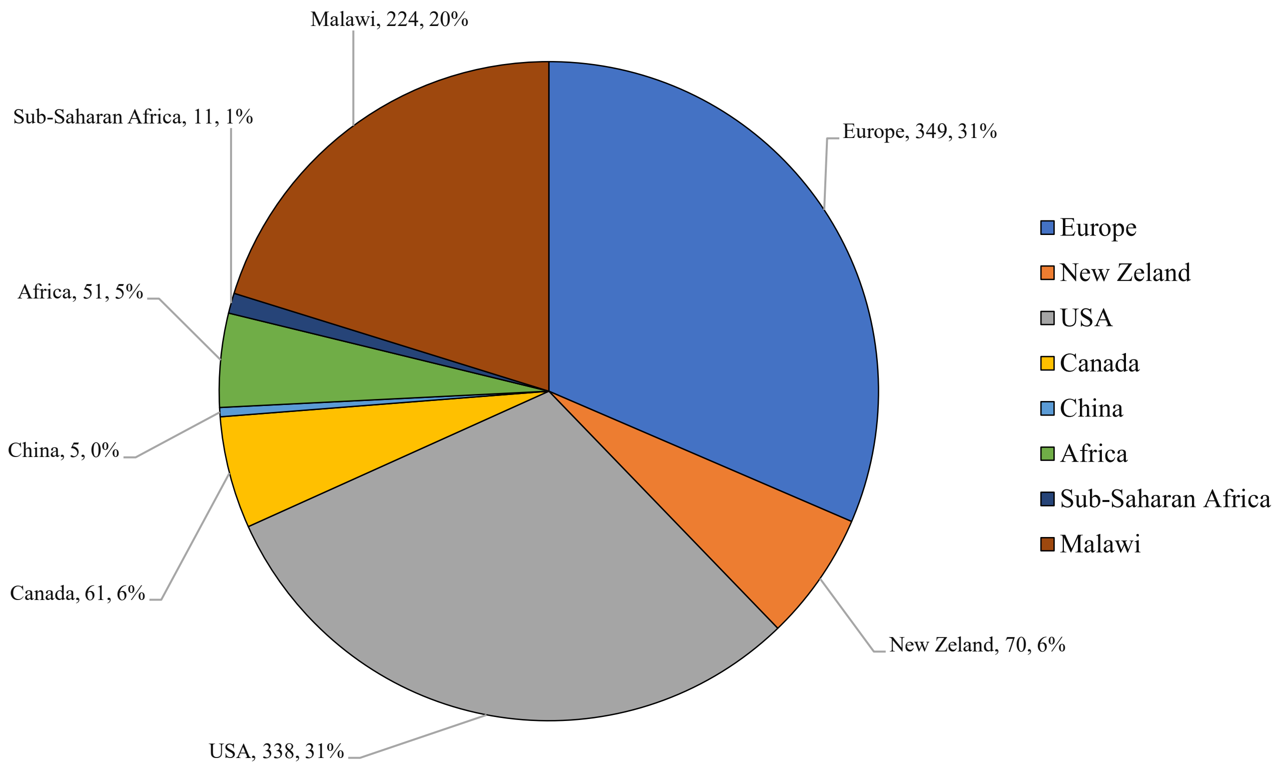

b)

Age

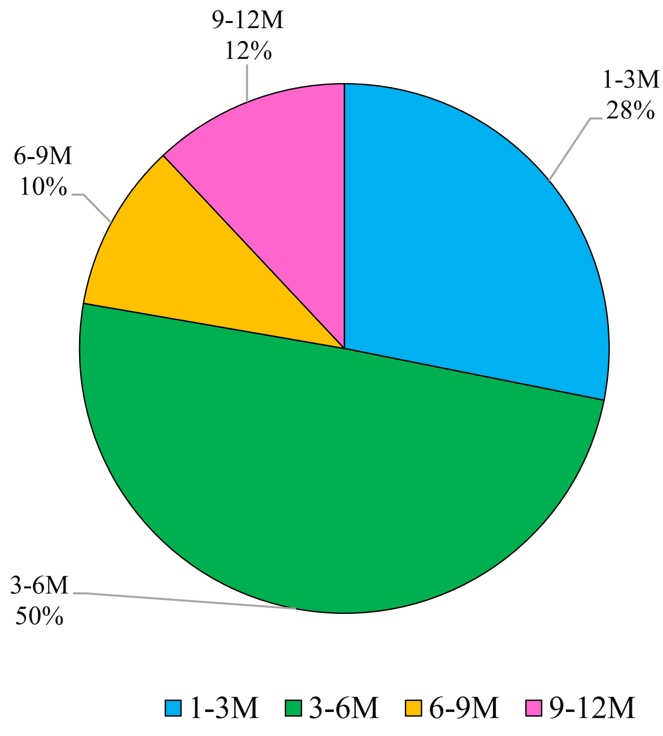

Cluster	Days Range	Months
**1-3M**	0 - 90	0 - 3
**3-6M**	91 - 180	3 - 6
**6-9M**	181 - 270	6 - 9
**9-12M**	271 - 360	9 - 12

Figure S1

Shallow shotgun microbial profiling at genera level of Côte d'Ivoire

a)

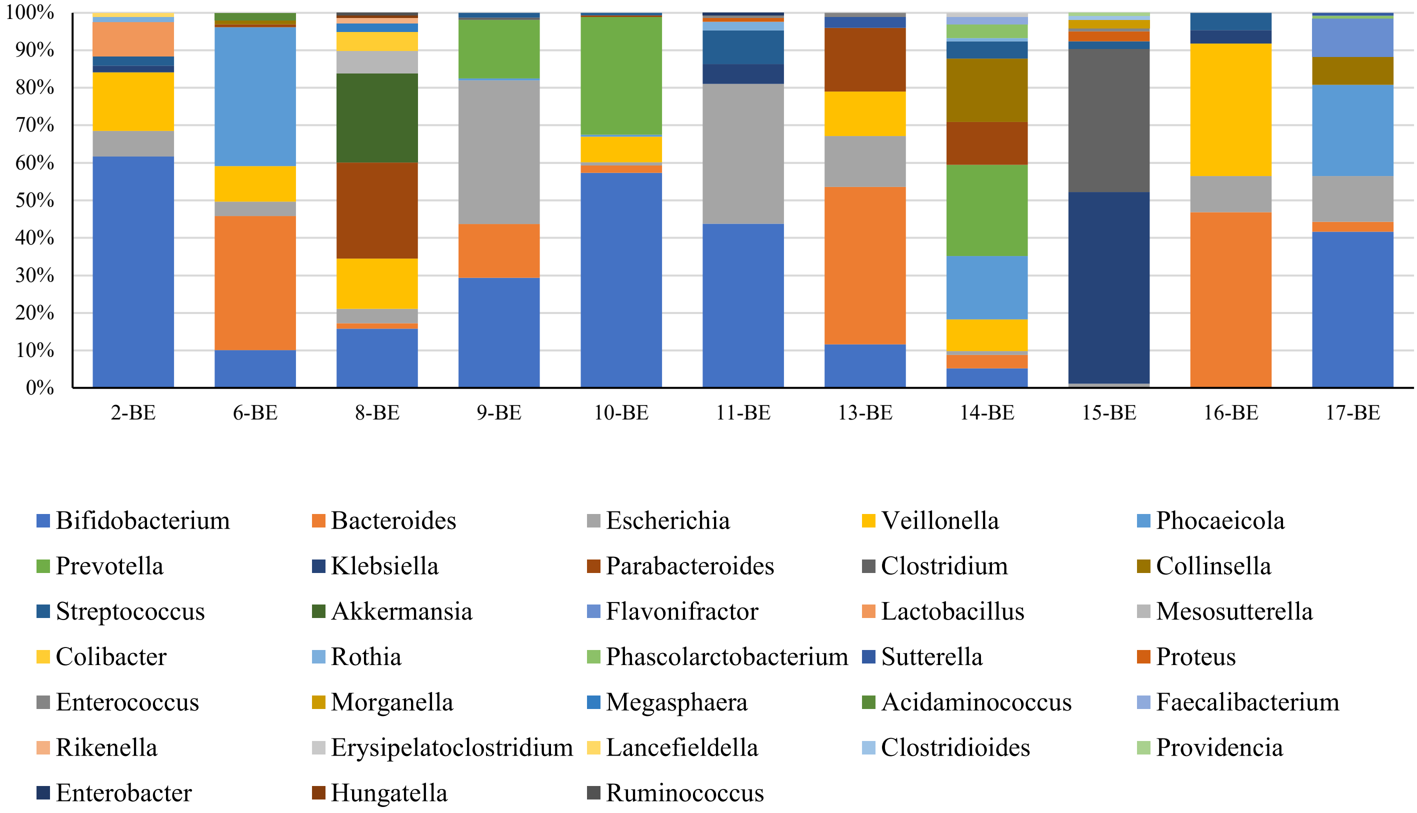

b)

16S rRNA gene microbial profiling at genera level of Côte d'Ivoire

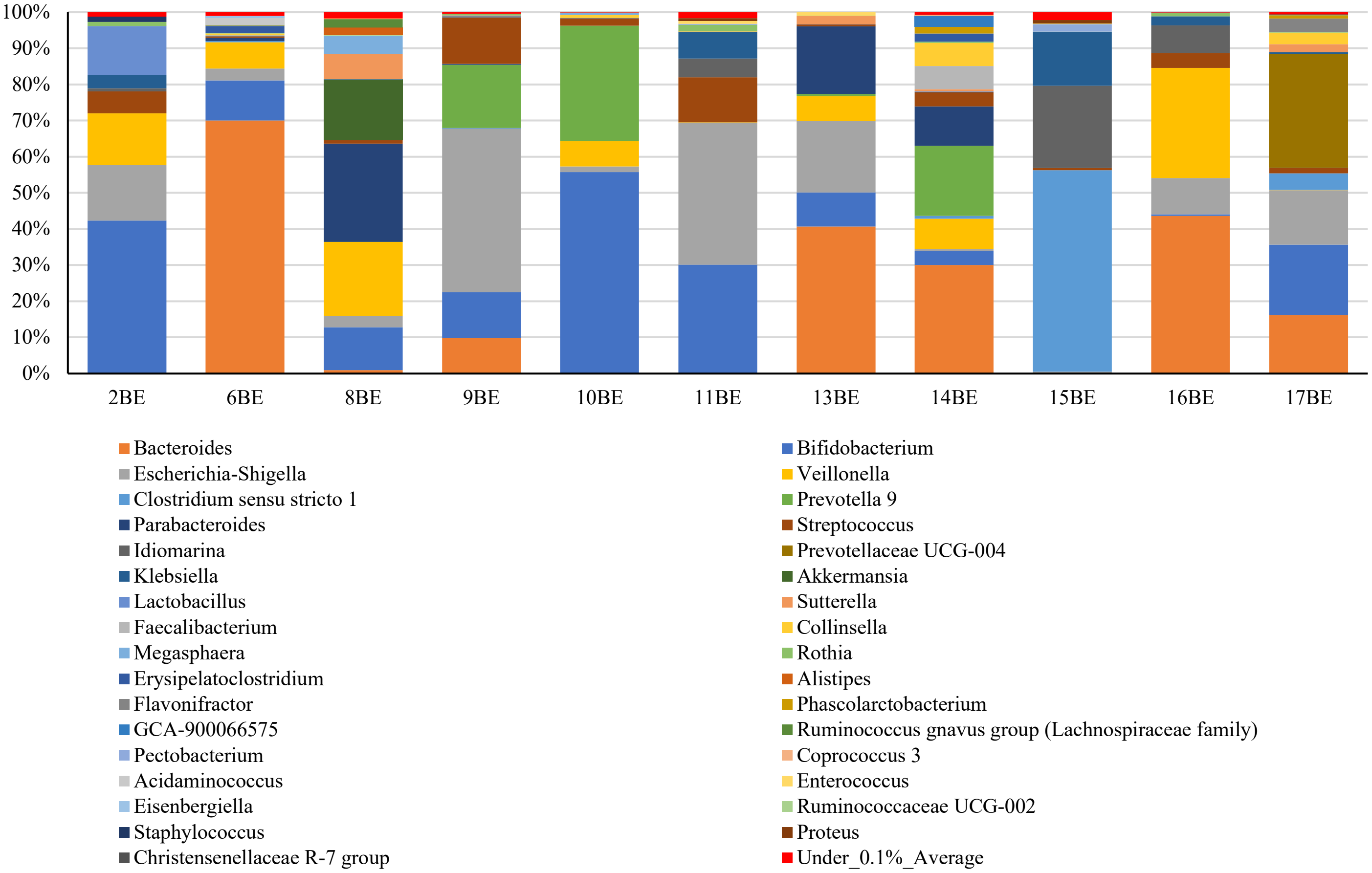

Figure S2

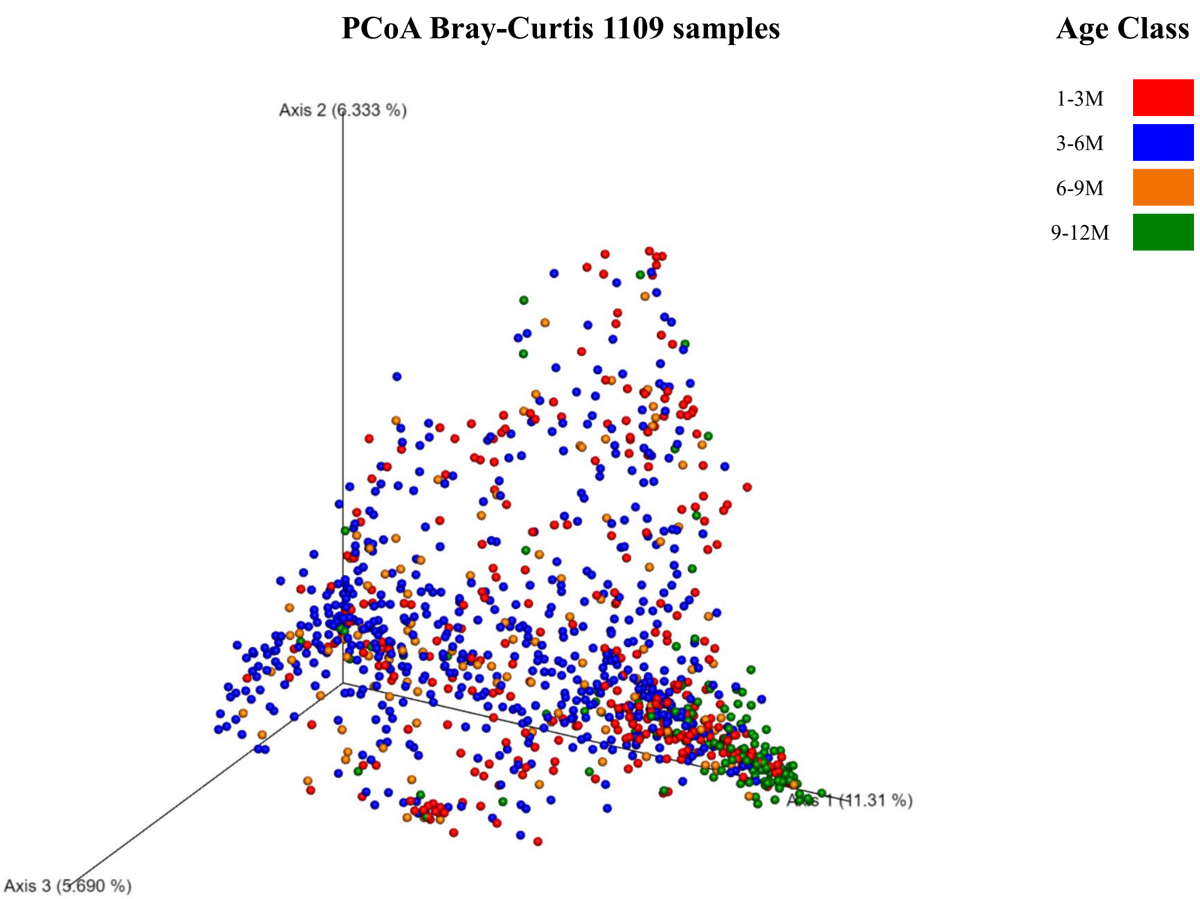
PCoA Bray-Curtis 1109 samples
Axis 2 (6.333 %)
Axis 1 (11.31 %)
Axis 3 (5.690 %)
Age Class
1-3M
3-6M
6-9M
9-12M

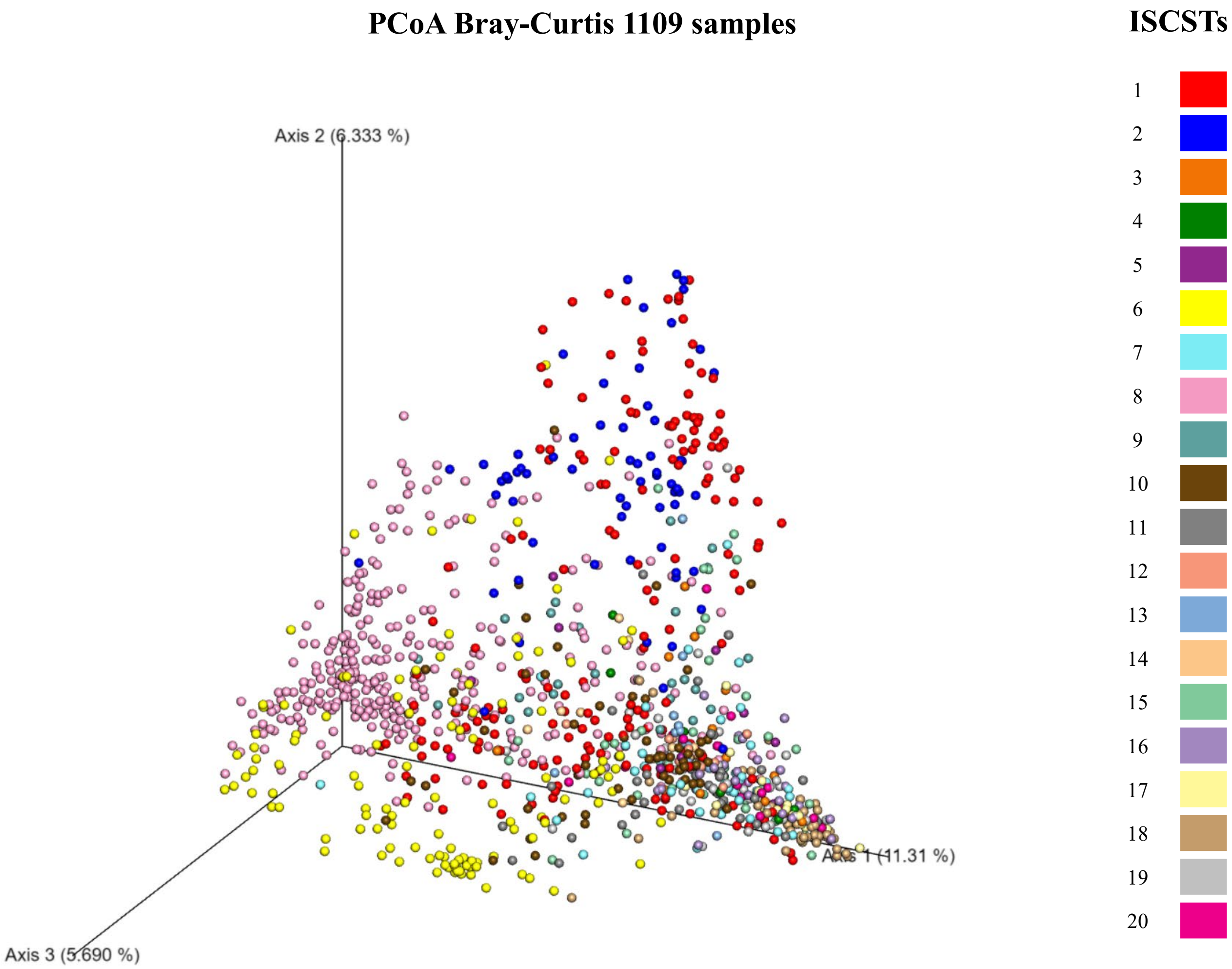
PCoA Bray-Curtis 1109 samples
Axis 2 (6.333 %)
Axis 1 (11.31 %)
Axis 3 (5.690 %)
ISCSTs
1
2
3
4
5
6
7
8
9
10
11
12
13
14
15
16
17
18
19
20

Figure S3

a)

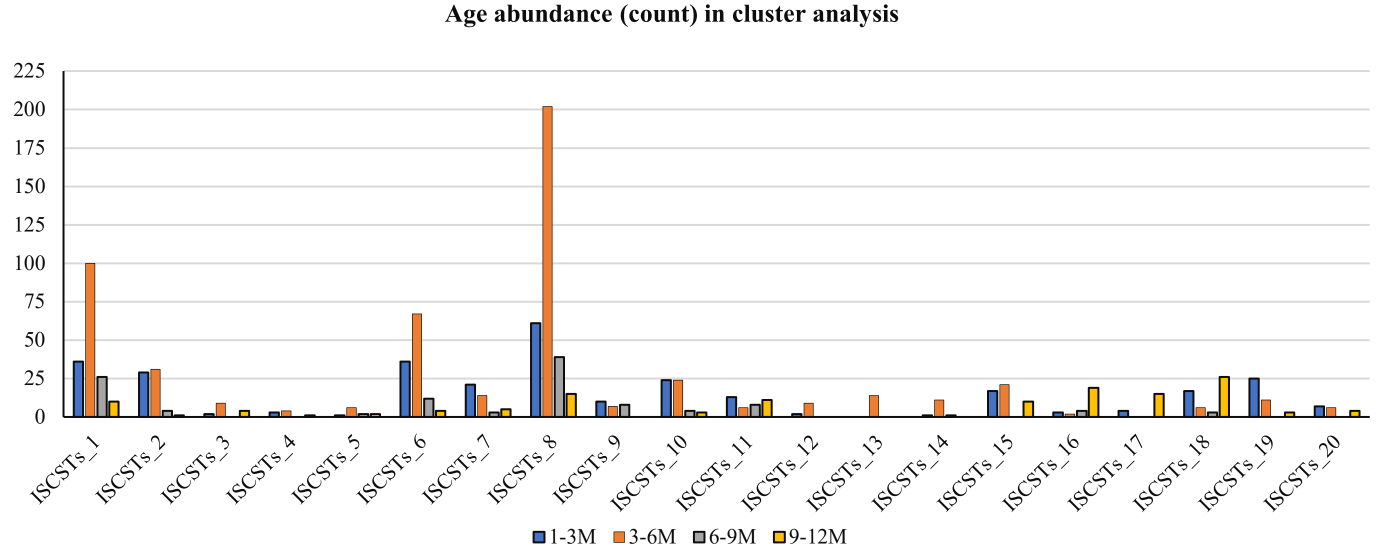

b)

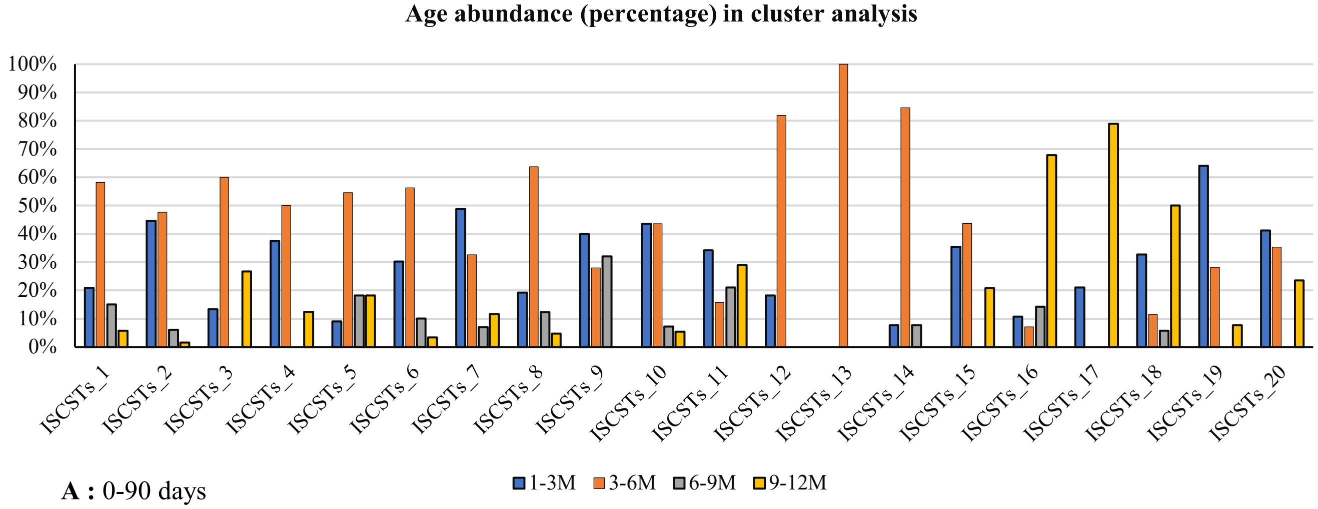

A : 0-90 days
B : 90-180 days
C : 180-270 days
D : 270-360 days

c)

				Age group count				Age group percentage			
**Alpha diversity (sample)**	**Species count**	**Samples count**	**ISCSTs_ID**	**A**	**B**	**C**	**D**	**A**	**B**	**C**	**D**
16.0	348	172	**ISCSTs_1**	36	100	26	10	20.93%	58.14%	15.12%	5.81%
12.3	214	65	**ISCSTs_2**	29	31	4	1	44.62%	47.69%	6.15%	1.54%
15.8	110	15	**ISCSTs_3**	2	9	0	4	13.33%	60.00%	0.00%	26.67%
17.6	87	8	**ISCSTs_4**	3	4	0	1	37.50%	50.00%	0.00%	12.50%
23.2	129	11	**ISCSTs_5**	1	6	2	2	9.09%	54.55%	18.18%	18.18%
12.8	281	119	**ISCSTs_6**	36	67	12	4	30.25%	56.30%	10.08%	3.36%
16.1	187	43	**ISCSTs_7**	21	14	3	5	48.84%	32.56%	6.98%	11.63%
15.6	389	317	**ISCSTs_8**	61	202	39	15	19.24%	63.72%	12.30%	4.73%
17.6	147	25	**ISCSTs_9**	10	7	8	0	40.00%	28.00%	32.00%	0.00%
15.9	230	55	**ISCSTs_10**	24	24	4	3	43.64%	43.64%	7.27%	5.45%
19.4	175	38	**ISCSTs_11**	13	6	8	11	34.21%	15.79%	21.05%	28.95%
19.7	120	11	**ISCSTs_12**	2	9	0	0	18.18%	81.82%	0.00%	0.00%
14.4	97	14	**ISCSTs_13**	0	14	0	0	0.00%	100.00%	0.00%	0.00%
20.5	131	13	**ISCSTs_14**	1	11	1	0	7.69%	84.62%	7.69%	0.00%
15.5	175	48	**ISCSTs_15**	17	21	0	10	35.42%	43.75%	0.00%	20.83%
23.9	157	28	**ISCSTs_16**	3	2	4	19	10.71%	7.14%	14.29%	67.86%
27.7	132	19	**ISCSTs_17**	4	0	0	15	21.05%	0.00%	0.00%	78.95%
24.1	208	52	**ISCSTs_18**	17	6	3	26	32.69%	11.54%	5.77%	50.00%
19.0	190	39	**ISCSTs_19**	25	11	0	3	64.10%	28.21%	0.00%	7.69%
24.5	153	17	**ISCSTs_20**	7	6	0	4	41.18%	35.29%	0.00%	23.53%

Figure S4

a)

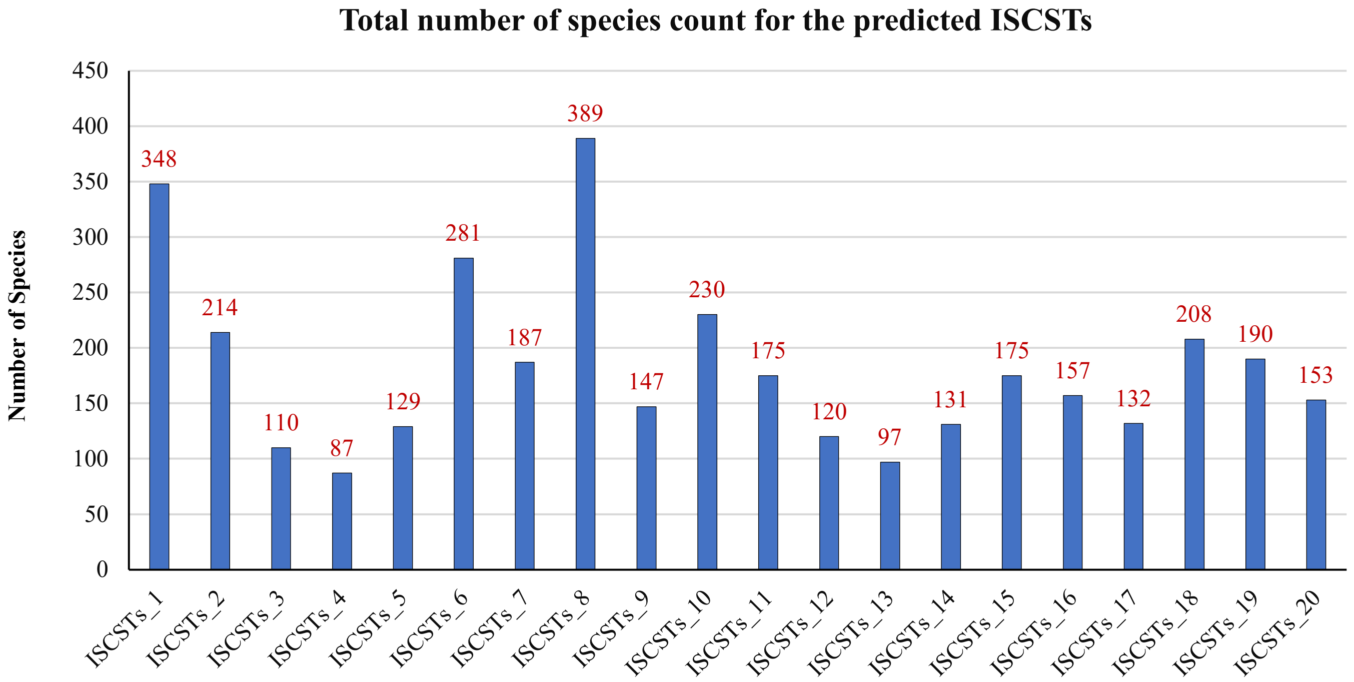
Total number of species count for the predicted ISCSTs
Number of Species
450
400
350
300
250
200
150
100
50
0
348
214
110
87
129
281
187
389
147
230
175
120
97
131
175
157
132
208
190
153
ISCSTs_1
ISCSTs_2
ISCSTs_3
ISCSTs_4
ISCSTs_5
ISCSTs_6
ISCSTs_7
ISCSTs_8
ISCSTs_9
ISCSTs_10
ISCSTs_11
ISCSTs_12
ISCSTs_13
ISCSTs_14
ISCSTs_15
ISCSTs_16
ISCSTs_17
ISCSTs_18
ISCSTs_19
ISCSTs_20

b)

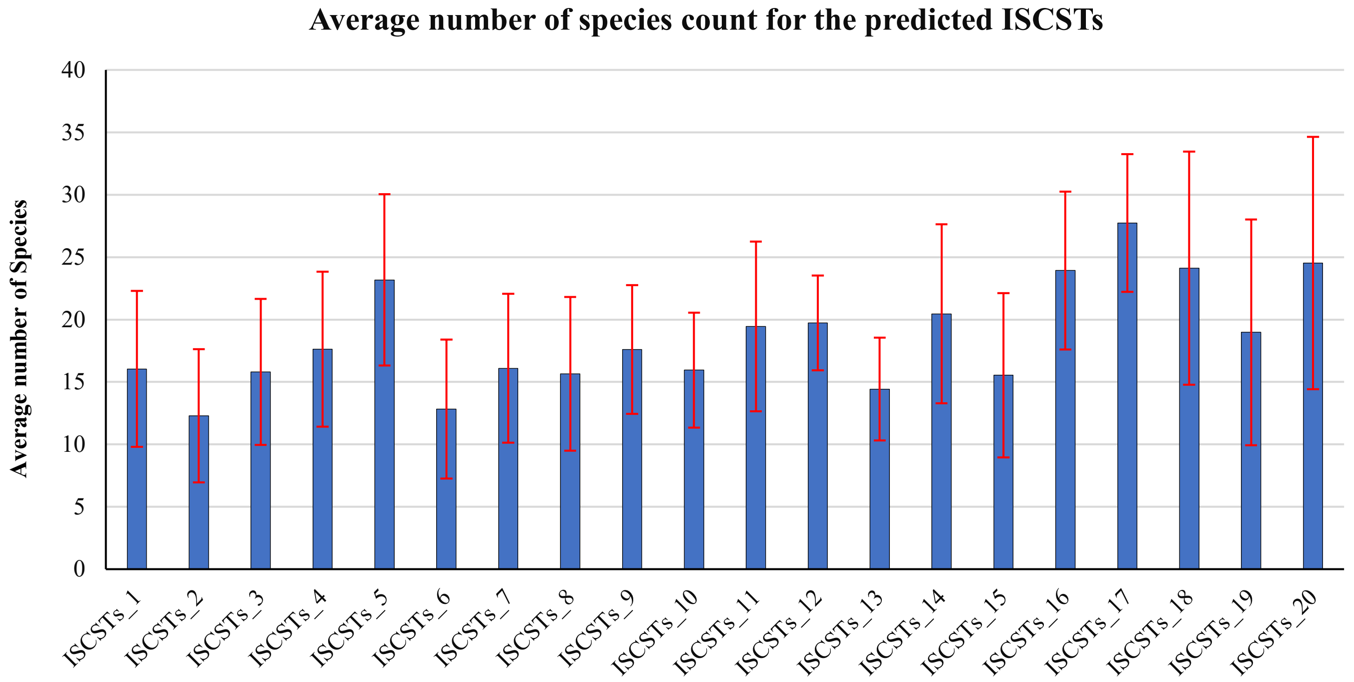
Average number of species count for the predicted ISCSTs
Average number of Species
40
35
30
25
20
15
10
5
0
ISCSTs_1
ISCSTs_2
ISCSTs_3
ISCSTs_4
ISCSTs_5
ISCSTs_6
ISCSTs_7
ISCSTs_8
ISCSTs_9
ISCSTs_10
ISCSTs_11
ISCSTs_12
ISCSTs_13
ISCSTs_14
ISCSTs_15
ISCSTs_16
ISCSTs_17
ISCSTs_18
ISCSTs_19
ISCSTs_20

Figure S5

a)

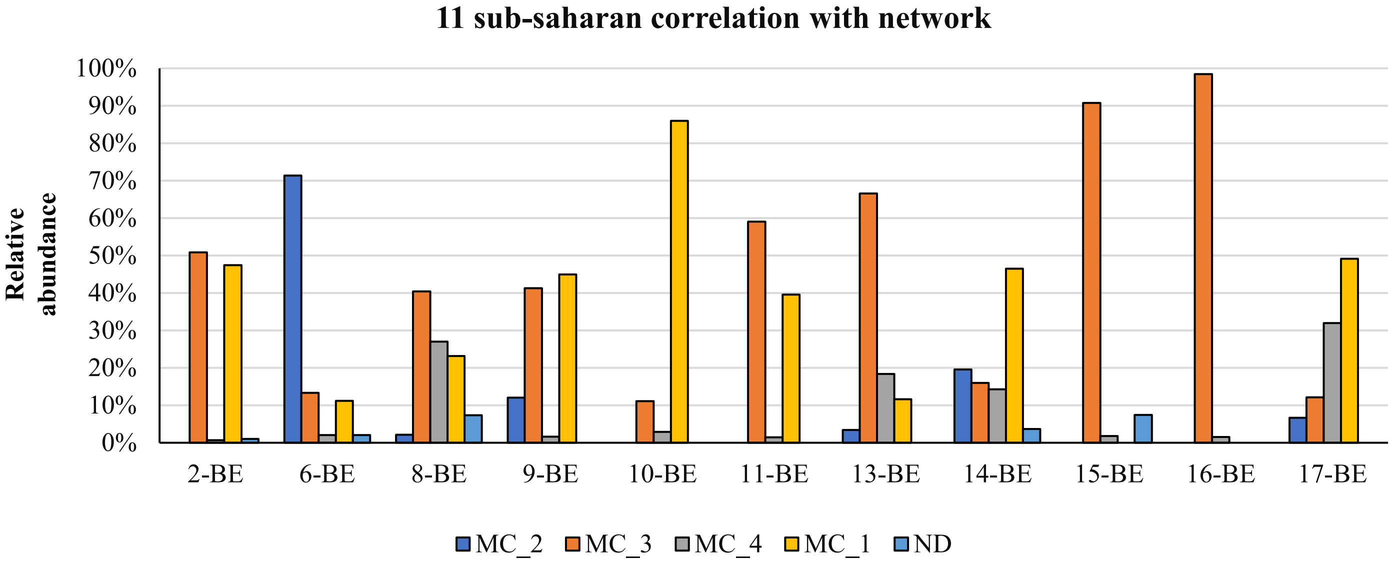

b)

Relative abundance

Modularity Class	2-BE	6-BE	8-BE	9-BE	10-BE	11-BE	13-BE	14-BE	15-BE	16-BE	17-BE
MC_2	0.00%	71.36%	2.11%	12.06%	0.00%	0.00%	3.43%	19.54%	0.00%	0.00%	6.71%
MC_3	50.83%	13.33%	40.42%	41.32%	11.13%	59.02%	66.55%	16.02%	90.77%	98.48%	12.14%
MC_4	0.68%	2.07%	26.97%	1.64%	2.88%	1.44%	18.41%	14.31%	1.80%	1.52%	31.98%
MC_1	47.46%	11.21%	23.12%	44.99%	85.99%	39.54%	11.61%	46.45%	0.00%	0.00%	49.17%
ND	1.03%	2.02%	7.37%	0.00%	0.00%	0.00%	0.00%	3.68%	7.43%	0.00%	0.00%

c)

Rural and Urban samples correlated to Modularity Cluster

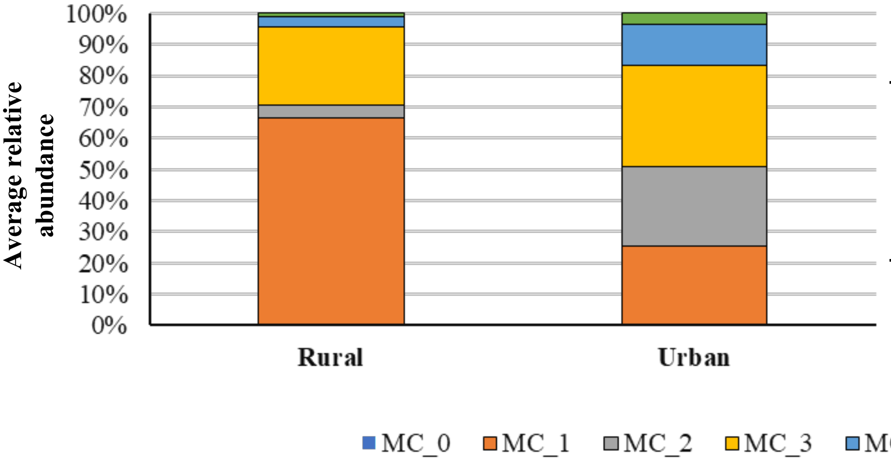

Average relative abundance

MC_ID	Rural	Urban
MC_1	66.26%	25.31%
MC_2	4.37%	25.64%
MC_3	25.16%	32.23%
MC_4	2.98%	12.96%
ND	1.23%	3.75%

Figure S6
